# Length of initial prescription at hospital discharge and long-term medication adherence for elderly, post-myocardial infarction patients: a population-based interrupted time series study

**DOI:** 10.1186/s12916-022-02401-5

**Published:** 2022-06-21

**Authors:** J. D. Schwalm, Noah M. Ivers, Zachary Bouck, Monica Taljaard, Madhu K. Natarajan, Francis Nguyen, Waseem Hijazi, Kednapa Thavorn, Lisa Dolovich, Tara McCready, Erin O’Brien, Jeremy M. Grimshaw

**Affiliations:** 1 Population Health Research Institute, McMaster University and Hamilton Health Sciences, DBCVSRI, 237 Barton Street East, Hamilton, ON L8L 2X2 Canada; 2 Division of Cardiology, Department of Medicine, McMaster University, Hamilton, ON Canada; 3 Family Practice Health Centre, Women’s College Hospital, Toronto, ON Canada; 4 Department of Family and Community Medicine, University of Toronto, Toronto, ON Canada; 5 ICES, Toronto, ON Canada; 6 Epidemiology Division, Dalla Lana School of Public Health, University of Toronto, Toronto, ON Canada; 7 Clinical Epidemiology Program, Ottawa Hospital Research Institute, University of Ottawa, Ottawa, ON Canada; 8 School of Epidemiology and Public Health, University of Ottawa, Toronto, ON Canada; 9 Department of Medicine, University of Ottawa, Ottawa, ON Canada; 10 Leslie Dan Faculty of Pharmacy, University of Toronto, Toronto, ON Canada

**Keywords:** Post-myocardial infarction, Adherence, Standardized discharge prescription form, Secondary prevention, Policy change

## Abstract

**Background:**

Preliminary evidence suggests that providing longer duration prescriptions at discharge may improve long-term adherence to secondary preventative cardiac medications among post-myocardial infarction (MI) patients. We implemented and assessed the effects of two hospital-based interventions—(1) standardized prolonged discharge prescription forms (90-day supply with 3 repeats for recommended cardiac medications) plus education and (2) education only—on long-term cardiac medication adherence among elderly patients post-MI.

**Methods:**

We conducted an interrupted time series study of all post-MI patients aged 65–104 years in Ontario, Canada, discharged from hospital between September 2015 and August 2018 with ≥ 1 dispensation(s) for a statin, beta blocker, angiotensin system inhibitor, and/or secondary antiplatelet within 7 days post-discharge. The standardized prolonged discharge prescription forms plus education and education-only interventions were implemented at 2 (1,414 patients) and 4 (926 patients) non-randomly selected hospitals in September 2017 for 12 months, with all other Ontario hospitals (*n* = 143; 18,556 patients) comprising an external control group. The primary outcome, long-term cardiac medication adherence, was defined at the patient-level as an average proportion of days covered (over 1-year post-discharge) ≥ 80% across cardiac medication classes dispensed at their index fill. Primary outcome data were aggregated within hospital groups (intervention 1, 2, or control) to monthly proportions and independently analyzed using segmented regression to evaluate intervention effects. A process evaluation was conducted to assess intervention fidelity.

**Results:**

At 12 months post-implementation, there was no statistically significant effect on long-term cardiac medication adherence for either intervention—standardized prolonged discharge prescription forms plus education (5.4%; 95% CI − 6.4%, 17.2%) or education only (1.0%; 95% CI − 28.6%, 30.6%)—over and above the counterfactual trend; similarly, no change was observed in the control group (− 0.3%; 95% CI − 3.6%, 3.1%). During the intervention period, only 10.8% of patients in the intervention groups received ≥ 90 days, on average, for cardiac medications at their index fill.

**Conclusions:**

Recognizing intervention fidelity was low at the pharmacy level, and no statistically significant post-implementation differences in adherence were found, the trends in this study—coupled with other published retrospective analyses of administrative data—support further evaluation of this simple intervention to improve long-term adherence to cardiac medications.

**Trial registration:**

ClinicalTrials.gov: NCT03257579, registered June 16, 2017

Protocol available at: https://pubmed.ncbi.nlm.nih.gov/33146624/.

**Supplementary Information:**

The online version contains supplementary material available at 10.1186/s12916-022-02401-5.

## Background

Clinical guidelines recommend long-term use of the following cardiac medication classes for secondary prevention in post-myocardial infarction (MI) patients: antiplatelets (aspirin plus a secondary antiplatelet); statins; beta blockers; and angiotensin system inhibitors (i.e., an angiotensin-converting enzyme [ACE] inhibitor or angiotensin receptor blocker [ARB]) [1–3]. These medications are associated with a relative risk reduction of 60% for major cardiovascular events [1, 2]. However, prior research has shown that post-discharge cardiac medication adherence often declines progressively over time [4–13]. Multiple studies have shown an increased risk of morbidity and mortality associated with medication non-adherence in patients with coronary artery disease [11–15].

Observational studies have demonstrated that post-MI patients receiving longer initial prescriptions have greater long-term adherence to cardiac medications [4, 16–18]. Providing longer prescriptions for post-MI patients at the time of discharge could address barriers to medication adherence including prescription refill burden and unintentional forgetfulness due to lack of habituation [19–21]. Unfortunately, health system policies promote shorter prescriptions, with compensation for up to a maximum of 5 refills in 1 year, likely contributing to the fact that only a minority of post-MI patients in Ontario–Canada’s most populous province–receive longer initial prescriptions that cover ≥ 90 days [4, 22, 23]. Therefore, while medication non-adherence is a complex issue, this relatively simple intervention could have significant implications at the population level.

In the present study, our objectives were to evaluate the effects of two hospital-based interventions implemented in Ontario, Canada, between September 2017 and August 2018—(1) standardized prolonged discharge prescription forms (with 90 days and 3 repeats per recommended cardiac medication class) in combination with on-site clinician education about the potential benefits of prolonged prescriptions and (2) on-site clinician education only—on long-term cardiac medication adherence among post-MI patients. We hypothesized that, in comparison to usual care (no intervention), implementation of the study interventions—which promote increased duration of initial discharge prescriptions—would result in a greater proportion of post-MI patients with long-term cardiac medication adherence over time.

## Methods

### Study design and setting

The Myocardial Infarction Prescription Adherence Duration (MIPAD) study used a non-randomized controlled interrupted time series design to evaluate whether long-term cardiac medication adherence among post-MI patients age ≥ 65 years in Ontario, Canada, discharged from hospital between 1 September 2015 and 31 August 2018 (24 months pre- and 12 months post-intervention) could be improved via clinician-targeted education alone or in combination with standardized prolonged discharge prescription forms for recommended cardiac medications. Our study protocol was published in November 2020 [24]. Study conduct and reporting were guided by quality criteria proposed by Ramsey et al and recommendations by Turner and colleagues for interrupted time series designs [25, 26].

### Interventions

We selected six sites across three hospital corporations within the same health region (the Hamilton Niagara Haldimand Brant region) in Ontario, Canada, to implement one of two non-randomly allocated interventions in September 2017 for 12 months. We included all remaining hospitals in Ontario that received no study intervention as a concurrent, external control group [24].

Both interventions are described fully in our protocol [24]. In brief, the first intervention was implemented at two hospital sites (one is a cardiac center, i.e., has a cardiac catheterization lab) within the same corporation and consisted of two components: (1) updated *standardized prolonged discharge prescription forms* featuring a default 90-day supply with 3 repeats for recommended secondary prevention cardiac medications, which were instituted in all wards where MI patients are managed, and (2) *education*, which involved regional educational rounds upon implementation to on-site clinicians promoting longer prescription durations (with quarterly email reminders thereafter), in addition to educational outreach (via personal emails and newsletters) to community pharmacies to encourage dispensation of prolonged discharge medications as prescribed. This intervention is referred to as “standardized prolonged discharge prescription forms plus education” hereafter. The second intervention, which was implemented at the other four selected sites (one cardiac center) across two distinct corporations, only involved the educational component (referred to as “education only” hereafter).

### Data sources

Data were obtained from population-based administrative databases that were linked using encoded identifiers and analyzed at ICES—a non-profit research institute authorized under Ontario’s health information privacy law to collect and analyze health care and demographic data for health system improvement and evaluation. The databases at ICES include information on demographics, comorbidities, and vital statistics for nearly all of Ontario’s 14.6 million residents, as well as all prescription medication dispensations covered under the province’s public drug funding system (the Ontario Drug Benefit [ODB] program), hospital discharges, emergency department visits, physician billing claims, and cardiac procedures in the province. Specific databases and their applications are detailed explicitly in subsequent sections. Use of ICES data for this study was authorized under section 45 of Ontario’s Personal Health Information Protection Act, which does not require review by a Research Ethics Board.

### Participants

The CorHealth Ontario Cardiac Registry captures demographics, comorbidities, and procedure-specific details (including coronary anatomy) for patients who undergo advanced cardiac procedures in Ontario and was used to identify all cardiac catheterization procedures completed between 2 August 2015 and 31 August 2018 for a primary reason of ST-elevated MI (STEMI) or non-ST-elevated MI (NSTEMI) with evidence of significant coronary artery disease (defined as left main artery stenosis ≥ 50% or major epicardial coronary stenosis ≥ 70%) [24]. We only retained cardiac catheterization records that met all of the following criteria: (1) performed on an Ontario resident with a valid provincial health card number; (2) non-missing patient discharge/transfer date; (3) patient aged ≥ 65 years (i.e., age-eligible for prescription drug coverage under the ODB plan) and < 105 years old; and (4) patient alive at discharge according to vital information on procedure record and the Registered Persons Database [24]. Restriction to patients aged 65 and older was done to ensure all participants dispensed cardiac medication prescriptions during the study period would be captured in the ODB database, since younger patients (< 65 years) pay for their medications out-of-pocket or through private insurance plans unless they qualify for ODB coverage through social support programs. Thus, we excluded patients < 65 years of age as we could not access dispensation records for their medications paid in cash or via private insurance. To minimize misclassification around the timing and location (hospital) from which a given patient was discharged, we attempted to link each unique catheterization record by procedure date with an overlapping episode of inpatient care for the same patient (according to Discharge Abstract Database claims) that ended with the patient being discharged home between 1 September 2015 and 31 August 2018. For patients with multiple catheterizations meeting the preceding eligibility criteria, only their first (earliest) catheterization was selected. Using the ODB database, we then restricted our study population to patients with a prescription dispensation claim for at least one recommended cardiac medication class of interest (statin, beta blocker, angiotensin system inhibitor, and/or secondary antiplatelet [i.e., prasugrel, ticagrelor, or clopidogrel]) at their first (“index”) fill within 7 days post-discharge [24]. We did not measure aspirin as it is available without a prescription and, as such, is rarely captured in the ODB database.

### Outcomes

#### Primary outcome

The primary outcome was long-term cardiac medication adherence measured at the patient level using prescription dispensation claims data from the ODB database [24]. For each patient, we identified the number of recommended cardiac medication classes (range, 1–4) dispensed at their index fill. Next, per dispensed class, we calculated the proportion of days covered (PDC) over 1 year from hospital discharge. If patients filled multiple prescriptions for different medications within the same class with overlapping days supplied, we assumed the supply was used in sequence [27]. For patients who died during follow-up, class-specific PDC values were calculated based on the time elapsed (in days) between discharge and death. Lastly, per patient, we divided the sum of class-specific PDC values by the number of dispensed classes at their index fill to derive that patient’s average PDC across classes. Long-term cardiac medication adherence was defined as an average PDC ≥ 80% [10].

#### Secondary outcomes

Several secondary patient-level outcomes were assessed. At index fill, we separately measured whether the duration (i.e., days supplied) was ≥ 90 days (considered a “prolonged dispensation”) for each dispensed cardiac medication class and whether the average initial duration was ≥ 90 days across all dispensed cardiac medication classes [24].

Additional medication-related secondary outcomes were measured including medication-class specific adherence (PDC ≥ 80%) and persistence (no period of ≥ 30 days without supply) at 1 year from discharge, as well as the number of cardiac medication classes dispensed to a patient at their index fill (range, 1–4) [24].

We also assessed the following health care utilization and adverse clinical outcomes at 1-year post-discharge: frequency of outpatient primary care visits; frequency of outpatient cardiology visits; time-to-hospitalization (in days) for (a) cardiovascular disease, (b) repeat acute MI, (c) stroke; time-to-hospitalization (in days) for (a) repeat cardiac catheterization and (b) coronary revascularization; and time-to-death (all-cause; in days) [24]. Visits or events occurring on the same day as discharge were not counted.

### Statistical analysis

#### Aggregate segmented regression analyses

Consistent with our published protocol [24], the main analysis of the primary outcome involved aggregating monthly data across all sites within an intervention group and analyzing the resulting monthly proportions (expressed as percentages) using segmented linear regression with first-order autoregressive errors (to account for autocorrelation) and fixed effects for time (in months; treated as continuous), intervention (1: post-implementation; 0: pre-implementation); and time after intervention (in months; treated as continuous). A group-month unit of analysis was chosen to facilitate expression of intervention effects on the absolute difference scale and promote time series stability [24]. Model parameters were estimated using restricted maximum likelihood estimation [28]. Estimated intervention effects are expressed as absolute changes in the level (intercept) and trend (slope) post-implementation with 95% confidence intervals (CI), which were respectively interpreted as the immediate and gradual effects of the intervention on long-term cardiac medication adherence. Additionally, we estimated the overall effect of each intervention at 12 months post-implementation, on the absolute scale, with 95% CI by comparing the fitted post-intervention outcome response and extrapolated outcome response based only on pre-intervention data (i.e., the counterfactual trend) at the end of the study.

An identical approach to pooling and analyzing outcome data at the group-month level was taken for (1) the primary outcome in the control group and (2) each secondary outcome measuring initial duration of ≥ 90 days at index fill (per class and on average) in the intervention and control groups.

#### Patient-level segmented regression analyses

We additionally analyzed all outcomes using segmented generalized linear regression with the patient (versus group-month) as the unit of analysis and terms for time, intervention, time after intervention, and the following patient-level covariates: age; sex; primary reason for cardiac catheterization (STEMI vs NSTEMI); prior MI; prior cardiac medication use (defined as ≥ 1 dispensation for any recommended cardiac medication class within 120 days before discharge); and site (fixed effect for intervention groups; random effect for control group) [23]. We did not adjust our patient-level segmented regression analysis according to the MI-treatment received (PCI [percutaneous coronary intervention], CABG [coronary artery bypass graft surgery], or medical therapy alone) as previously published work using the same data sources, demonstrated no difference in medication adherence in these three groups [4]. For dichotomous outcomes, we specified a binary distribution with logit link and expressed intervention effects as odds ratios with 95% CI; for count-based outcomes, we specified a negative binomial distribution with log link and expressed intervention effects as rate ratios with 95% CI; and for time-to-event outcomes, we used Cox proportional hazards regression models and expressed intervention effects as hazard ratios with 95% CI. For non-fatal, time-to-event outcomes, death was treated as a censoring event [24].

### Process evaluation

A process evaluation was undertaken to assess intervention fidelity at the (1) provider level (using the standardized prescriptions where applicable and prescribing ≥ 90 days with ≥ 3 repeats for cardiac medications) and (2) at the pharmacy level (dispensing the prescribed amount for cardiac medication prescriptions). Using local CorHealth Ontario Cardiac registry data and the approach described in the “Participants” section, we identified 731 eligible post-MI patients discharged from an intervention site during the 12-month implementation period: 484 from the standardized prolonged discharged prescription forms plus education intervention group (2/2 sites represented) and 247 from the education-only intervention group (2/4 sites represented). We excluded patients from the other two education-only sites due to limitations in accessing discharge prescription records at these sites. We randomly sampled 65 patients per intervention group and attempted to link their catheterization claim with their discharge hospital chart data. Among linked patients, we restricted our analysis to patients with ≥ 1 eligible cardiac medication discharge prescription.

Within each intervention group, hospital chart data were used to calculate the proportion of sampled patients with a prolonged discharge prescription (i.e., ≥ 90 days supplied with ≥ 3 repeats) during the intervention period for one of the four cardiac medication classes of interest. Additionally, we calculated the proportion of individuals with an average duration (i.e., days supplied per fill) of ≥ 90 days across all prescribed cardiac medication classes without consideration of the number of repeats. For comparison with our chart-based results, we also present the observed proportion of patients who, at their index fill, had a prolonged initial dispensation (i.e., ≥ 90 days supplied) per class and an average duration (i.e., days supplied) ≥ 90 days across all dispensed cardiac medication classes based on ODB data from the intervention period. These comparisons were intended to help us assess the level of agreement between discharge prescription duration and initial pharmacy dispensation duration. Additionally, we summarized the frequency (and proportion) of patients who received the standardized prolonged discharged prescription form as planned.

All analyses were conducted using SAS Version 9.4 (SAS Institute Inc.; Cary, NC).

## Results

### Study population

**Fig. 1:**
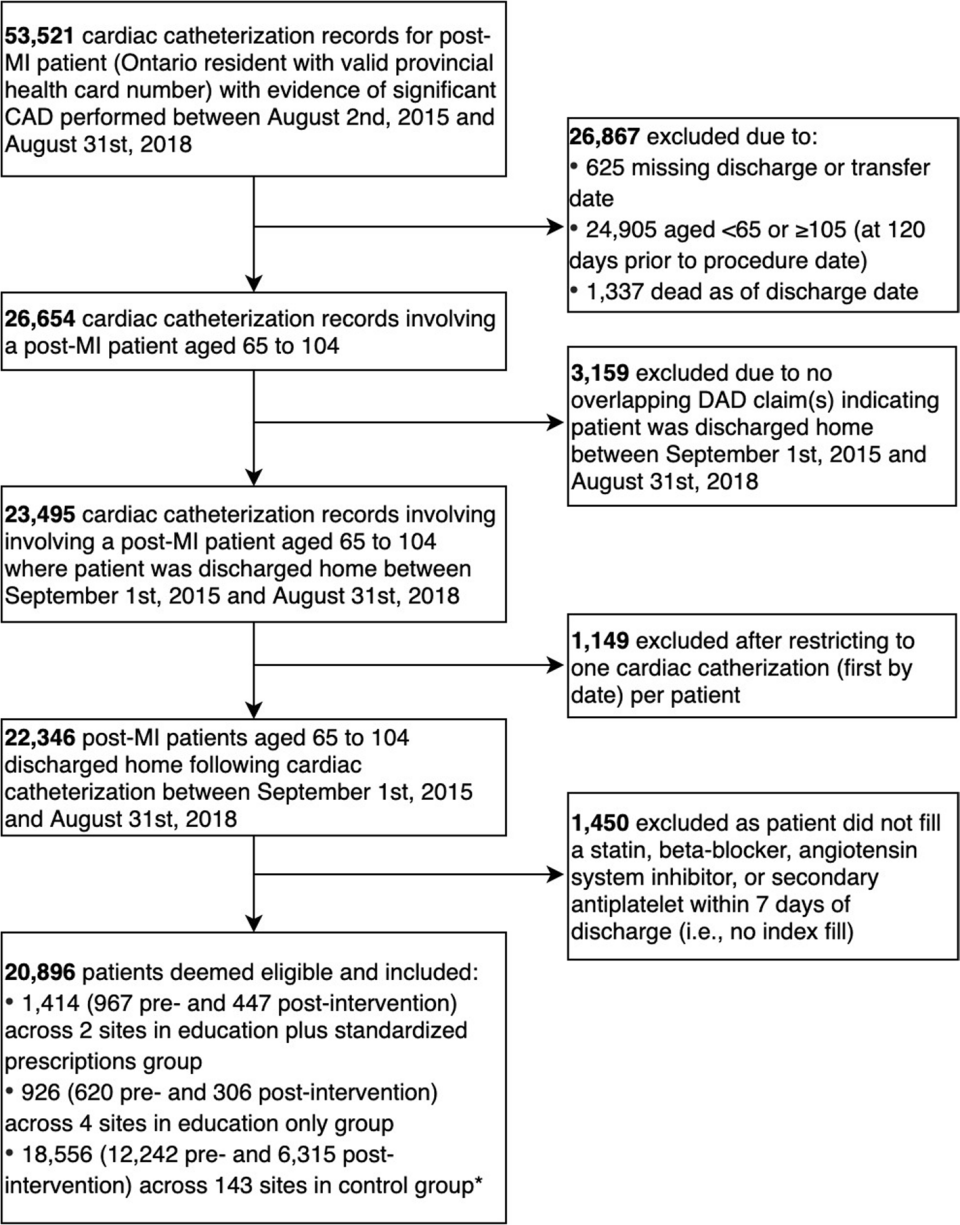
Flow of participants into the study. *Notes*: CAD = coronary artery disease; MI = myocardial infarction; DAD = Discharge Abstract Database. *For patient-level analyses, a threshold-based exclusion (i.e., minimum 180 eligible patients per site) was applied to facilitate model convergence, resulting in 14,344 patients (9,429 pre- and 4,915 post-intervention) across 26 sites in the control group

Between 1 September 2015 and 31 August 2018, 20,896 eligible post-MI patients across 149 hospitals were discharged following a cardiac catheterization in Ontario (Fig. 1). Additional file 1: Table S1 describes baseline patient characteristics. On average, the monthly denominator consisted of 39 (range, 28–48), 26 (range, 14–36), and 515 (range, 439–596) patients in the standardized prolonged discharge prescription forms plus education, education-only, and control groups, respectively.

### Aggregate segmented regression analyses

#### Primary outcome

**Fig. 2:**
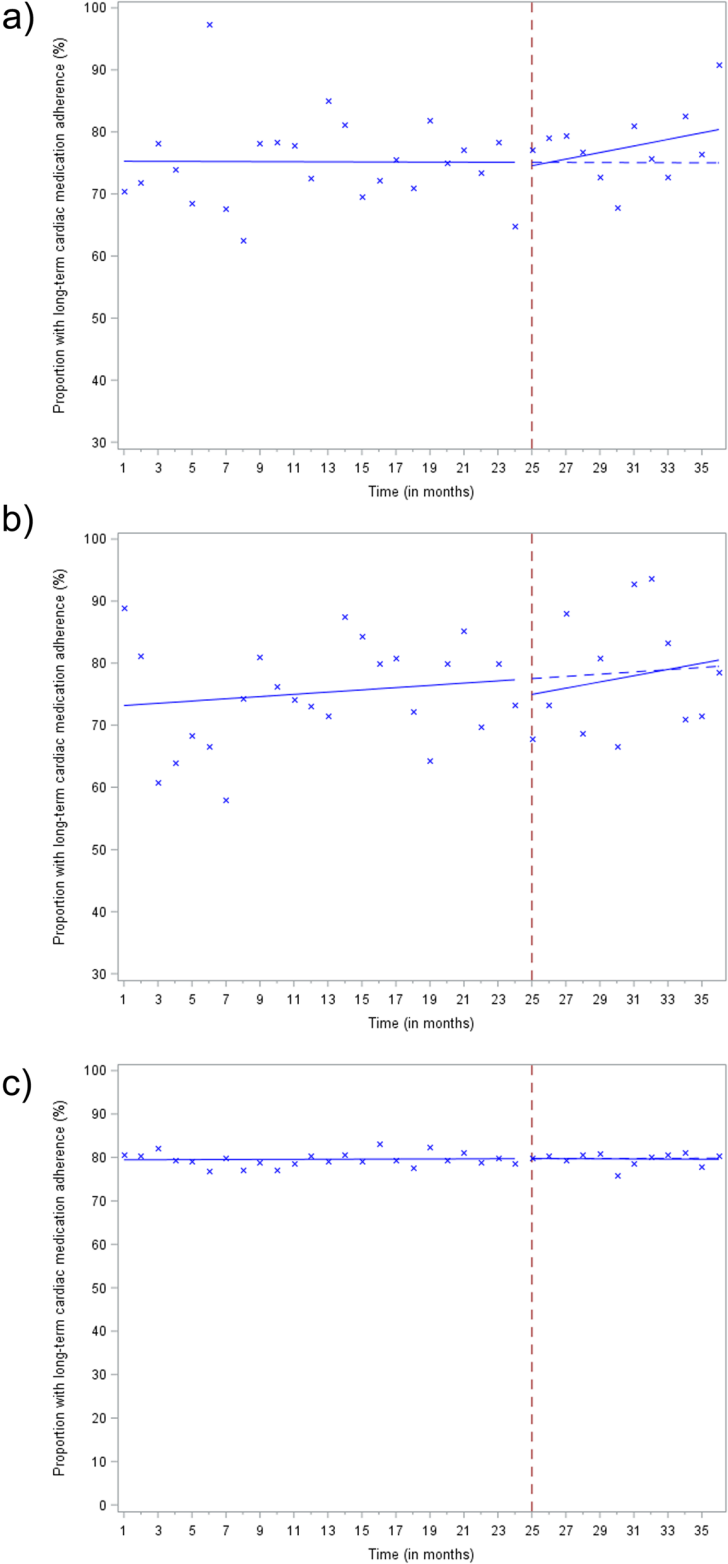
Monthly proportion of post-myocardial infarction patients age 65 and older with long-term cardiac medication adherence at one year from hospital discharge from September 2015 to August 2018 in Ontario, Canada by intervention group: **a**) Standardized prolonged discharge prescription forms plus education, (2 sites, 1414 patients); **b**) education only (4 sites, 926 patients); **c**) control (143 sites, 18556 patients). Notes: Observed values are denoted by ‘x’, solid blue lines represent the fitted regression pre- and post-intervention trendlines, and the hatched blue line represents the projected pre-intervention trend assuming there was no intervention (i.e., the counterfactual)

Table 1 presents estimated coefficients with 95% CI from the aggregate segmented linear regression analyses used, in part, to generate Fig. 2—which visualizes the observed time series for the monthly proportion of patients with long-term cardiac medication adherence by intervention group, along with the fitted pre- and post-implementation trends, and the extrapolated pre-implementation (i.e., counterfactual) trends.

**Table 1:** Results from aggregate segmented linear regression analyses estimating the absolute immediate, gradual, and overall (at 12 months post-intervention) effects of study interventions on long-term cardiac medication adherence (assessed at 1 year from hospital discharge) among post-myocardial infarction patients age 65 and older in Ontario, Canada, from September 2015 to August 2018

Parameter	Estimate, % (95% CI)	*P* value
Standardized prolonged discharge prescription forms plus education (2 sites, 1414 patients)		
Intercept (baseline percentage)	75.3 (70.1 to 80.4)	<.001
Pre-intervention slope (secular trend, per month)	− 0.01 (− 0.39 to 0.38)	.97
Change in level post-intervention (immediate effect)	− 1.08 (− 10.7 to 8.50)	.81
Change in trend post-intervention (gradual effect, per month)	0.54 (− 0.62 to 1.70)	.32
Overall effect at 12 months post-intervention (combined immediate and gradual effect)	5.36 (− 6.44 to 17.2)	.34
Education only (4 sites, 926 patients)		
Intercept (baseline percentage)	73.2 (60.0 to 86.4)	<.001
Pre-intervention slope (secular trend, per month)	0.18 (− 0.76 to 1.12)	.65
Change in level post-intervention (immediate effect)	− 2.87 (− 21.1 to 15.3)	.74
Change in trend post-intervention (gradual effect, per month)	0.32 (− 2.31 to 2.96)	.78
Overall effect at 12 months post-intervention (combined immediate and gradual effect)	1.01 (− 28.6 to 30.6)	.93
Control group (143 sites, 18556 patients)		
Intercept (baseline percentage)	79.5 (78.0 to 80.9)	<.001
Pre-intervention slope (secular trend, per month)	0.01 (− 0.10 to 0.12)	.83
Change in level post-intervention (immediate effect)	0.05 (− 2.59 to 2.70)	.97
Change in trend post-intervention (gradual effect, per month)	− 0.03 (− 0.35 to 0.30)	.87
Overall effect at 12 months post-intervention (combined immediate and gradual effect)	− 0.26 (− 3.64 to 3.12)	.89

*CI* confidence interval. All results based on group-specific, aggregate segmented linear regression models, which accounted for serial correlation in monthly time series data through first-order autoregressive (i.e., AR(1)) errors. AR(1) parameter estimates for each group-specific model were − 0.14, 0.34, and 0.01, respectively. Model parameters were estimated using restricted maximum likelihood estimation with the Satterthwaite adjustment for computing denominator degrees of freedom. Model MSE values were 46.1, 90.1, and 2.73, respectively

Implementation of the standardized prolonged discharge prescription forms plus education intervention was associated with an immediate, non-statistically significant decrease of 1.08 fewer adherent patients per 100 (*P* = .81), and a non-significant increase of 0.54 additional adherent patients per 100 per month (*P* = .32) above the underlying pre-intervention trend. At 12 months post-implementation, the standardized prolonged discharge prescription forms plus education intervention was associated with a statistically insignificant increase of 5.36 additional adherent patients per 100 (95% CI − 6.44 to 17.2) compared to if the intervention had never been implemented in this group.

Implementation of the education-only intervention was not associated with a statistically significant immediate (*P* = .74) or gradual (*P* = .78) change in long-term cardiac medication adherence. Similarly, at 12 months post-implementation, there was no statistically significant difference in adherence relative to the underlying counterfactual trend (1.01 additional adherent patients per 100, 95% CI − 28.6 to 30.6).

In the control group, we found no immediate (*P* = .97) or gradual change (*P* = .87) in long-term cardiac medication adherence following implementation of study interventions at the other six sites. Additionally, no statistically significant difference in long-term adherence was observed at 12 months post-implementation in the control group (0.26 fewer adherent patients per 100, 95% CI − 3.64 to 3.12).

#### Secondary outcomes

Instability in the group-specific monthly series for outcomes measuring initial durations for specific cardiac medication classes at index fill precluded modeling (results not shown). The results of our aggregate segmented linear regression analyses for the monthly proportion of patients with an average initial duration of ≥ 90 days across dispensed cardiac medication classes at index fill are summarized in Table 2 and visualized in Additional file 2: Fig. S1.

**Table 2:** Results from aggregate segmented linear regression analyses estimating the absolute immediate, gradual, and overall (at 12 months post-intervention) effects of study interventions on receiving an average initial duration of ≥ 90 days across cardiac medications dispensed at index fill among post-myocardial infarction patients age 65 and older in Ontario, Canada from September 2015 to August 2018

Parameter	Estimate, % (95% CI)	*P* value
Standardized prolonged discharge prescription forms plus education (2 sites, 1414 patients)		
Intercept (baseline percentage)	4.76 (− 1.28 to 10.8)	.10
Pre-intervention slope (secular trend, per month)	0.04 (− 0.40 to 0.47)	.83
Change in level post-intervention (immediate effect)	4.02 (− 5.15 to 13.2)	.35
Change in trend post-intervention (gradual effect, per month)	0.18 (− 1.06 to 1.43)	.73
Overall effect at 12 months post-intervention (combined immediate and gradual effect)	6.19 (− 7.51 to 19.9)	.30
Education only (4 sites, 926 patients)		
Intercept (baseline percentage)	6.16 (2.25 to 10.1)	.006
Pre-intervention slope (secular trend, per month)	0.10 (− 0.19 to 0.39)	.47
Change in level post-intervention (immediate effect)	3.24 (− 3.89 to 10.4)	.34
Change in trend post-intervention (gradual effect, per month)	− 0.48 (− 1.35 to 0.39)	.25
Overall effect at 12 months post-intervention (combined immediate and gradual effect)	− 2.55 (− 11.5 to 6.38)	.54
Control group (143 sites, 18556 patients)		
Intercept (baseline percentage)	7.40 (6.63 to 8.17)	<.001
Pre-intervention slope (secular trend, per month)	− 0.02 (− 0.07 to 0.04)	.57
Change in level post-intervention (immediate effect)	− 0.10 (− 1.52 to 1.31)	.88
Change in trend post-intervention (gradual effect, per month)	− 0.01 (− 0.18 to 0.16)	.89
Overall effect at 12 months post-intervention (combined immediate and gradual effect)	− 0.23 (− 2.00 to 1.54)	.78

*CI* confidence interval. All results based on group-specific, aggregate segmented linear regression models, which accounted for serial correlation in monthly time series data through first-order autoregressive (i.e., AR(1)) errors. AR(1) parameter estimates for each group-specific model were 0.22, − 0.07, and − 0.07, respectively. Model parameters were estimated using restricted maximum likelihood estimation with the Satterthwaite adjustment for computing denominator degrees of freedom. Model MSE values were 23.4, 22.3, and 0.88, respectively

Index fill defined as a patient’s first prescription fill within 7 days from hospital discharge, at which point ≥ 1 prescriptions were filled for a statin, beta blocker, angiotensin system inhibitor, and/or secondary antiplatelet

Implementation of the standardized prolonged discharge prescription forms plus education intervention was not associated with statistically significant immediate (*P* = .35) or gradual (*P* = .73) changes in the monthly proportion of patients receiving an average initial duration of ≥ 90 days across dispensed cardiac medications. At 12 months post-implementation, the standardized prolonged discharge prescription forms plus education intervention was associated with a non-statistically significant increase of 6.19 additional patients receiving an average initial duration of ≥ 90 days for dispensed cardiac medications (95% CI − 7.51 to 19.9) beyond the counterfactual trend.

Implementation of the education-only intervention did not correspond with statistically significant immediate (*P* = .34) or gradual (*P* = .25) changes in the monthly proportion of patients receiving an average initial duration of ≥ 90 days for dispensed cardiac medications. At 12 months post-implementation, the education-only intervention was associated with 2.55 fewer patients receiving an average initial duration of ≥ 90 days for dispensed cardiac medications (95% CI − 11.5 to 6.38) over and above the counterfactual trend.

In the control group, the implementation of the interventions at non-control sites was not associated with immediate (*P* = .88), gradual (*P* = .89), or overall (at 12 months post-implementation [relative to counterfactual trend]; *P* = .78) changes in the monthly proportion of patients in the control group receiving an average initial duration of ≥ 90 days for dispensed cardiac medications.

### Patient-level segmented regression analyses

Additional file 3: Table S2 and Additional file 4: Table S3 summarize patient-level segmented regression analysis results for primary and secondary outcomes. The results suggest that neither intervention had immediate or gradual effects on patients’ odds of long-term cardiac medication adherence or receiving initial prescription dispensations of prolonged duration (≥ 90 days). Similarly, our results pertaining to outcomes measuring health care utilization (expressed as counts) and adverse events (time-to-event) do not suggest intervention effects (on the rate ratio and hazard ratio scales, respectively) over and above secular trends observed in the external control group.

### Process evaluation

Among the randomly selected 130 patients (65 per intervention group) during the 12-month intervention period, 92 (71%) were successfully linked to their discharge hospital chart data and had ≥ 1 discharge prescription for a cardiac medication of interest. Of these patients, 41 were discharged from a site in the standardized prolonged discharge prescription forms plus education group and 51 were discharged from a site in the education-only group. The standardized prolonged discharge prescription form was only used for 44% (18/41) of sampled patients from that intervention group. Overall, 83% (34/41) and 67% (34/51) of patients in the standardized prolonged discharge prescription forms plus education and education-only groups were prescribed discharge cardiac medications with an average duration of ≥ 90 days per fill in the intervention period (Additional file 5: Table S4).

Table 3 summarizes group-specific proportions of patients who, at index fill, received a prolonged dispensation (i.e., ≥ 90 days supplied) at their local pharmacy per medication class and an average duration of ≥ 90 days per dispensed medication class (according to prescription dispensation records). In the 12-month intervention period, only 12% (53/447) and 9.5% (29/306) of post-MI patients ≥ 65 years old from the standardized prolonged discharge prescription forms plus education and education-only groups had an average duration of ≥ 90 days across their initial cardiac medication dispensations post-discharge.

**Table 3:** Proportion of patients with a prolonged dispensation (≥ 90 days supplied) at index fill by cardiac medication class according to Ontario Drug Benefit claims, stratified by intervention group and study period (pre- vs post-intervention)

	Standardized prolonged discharge prescription forms plus education		Education only	
	Pre-intervention	Post-intervention	Pre-intervention	Post-intervention
Average (across classes)^a^	50/967 (5.2%)	53/447 (12%)	46/620 (7.4%)	29/306 (9.5%)
Statins	132/775 (17%)	99/371 (27%)	97/517 (19%)	49/244 (20%)
Beta blockers	69/694 (9.9%)	52/322 (16%)	62/464 (13%)	31/223 (14%)
Angiotensin system inhibitors	73/501 (15%)	49/230 (21%)	53/356 (15%)	27/178 (15%)
Secondary antiplatelets	48/658 (7.3%)	44/326 (13%)	40/518 (7.7%)	27/266 (10%)

^a^ ≥ 90 days supplied on average across cardiac medication classes at index fill

## Discussion

To our knowledge, this is the first non-randomized interventional study to investigate how longer initial discharge prescription durations can influence medication adherence. Despite limited intervention fidelity according to process evaluation results, particularly at the pharmacy-level, we observed a 5.4% absolute increase in long-term medication adherence after 12 months post-implementation in the standardized prolonged discharge prescription forms plus education group. While this absolute difference was not statistically significant (95% CI − 6.44 to 17.2), it might be clinically important given the ease of implementation and low cost of the intervention. Further, this study highlights several important issues with respect to novel methodologies, use of individual cardiac medication classes post-MI, and policies potentially limiting the intervention fidelity.

High-quality reviews have outlined multiple barriers to medication adherence in chronic conditions, as well as interventions to mitigate these barriers at patient, health care provider, and health system levels [29–31]. Though non-adherence is a multifactorial issue, complex interventions that target multiple barriers are not very effective and difficult to sustain [30]. Therefore, evaluation of a simple health system-level intervention with even a modest impact but applied at the population level could result in significant improvements in clinical outcomes.

This simple, non-randomized intervention study was evaluated using pre-existing, routinely collected administrative data. All eligible participants in a province of over 14 million people were included in the analysis, with the vast majority discharged from a hospital in the control group. Given the limitations of the ODB database, only patients ≥ 65 years of age were included, despite the intervention being applied to all post-MI patients discharged from an intervention site during the implementation period. Therefore, given the limited number of intervention sites and age restrictions, only a fraction of eligible patients who received an intervention (standardized prolonged discharge prescription forms plus education or education-only) were analyzed. Although the findings in the combined intervention group were not statistically significant, the observed trends in long-term cardiac medication adherence over time within the intervention and control groups supports that the intervention may in fact have some value. Furthermore, the incremental improvements in long-term adherence are consistent with the varying intensity of the interventions. Specifically, at 12 months post-implementation, long-term medication adherence did not change at all in the provincial control group (0%), whereas adherence increased slightly in the education-only group (1%) and by a larger margin in the standardized prolonged discharge prescription forms plus education group (5%).

Poor intervention fidelity may have impeded the full potential for improved medication adherence in the intervention groups. As evidenced by our process evaluation, the fidelity of the intervention implementation was limited both at discharge and at the time of initial prescription fill. At discharge, only 44% of patients in the standardized prolonged discharge prescription forms plus education group received the revised study prescription as intended. The two hospital sites that implemented this two-pronged intervention used paper-based prescriptions. This format allows for deviations in prescribing practices (i.e., use of personal prescription pads or altering the standardized forms) and likely accounts for the suboptimal use of the standardized prescription forms. Despite the limited use of the standardized prescription forms, patients still received prescriptions with orders for cardiac medications to be dispensed for 90 days with repeats 83% of the time. In the education-only group, 67% of patients received prolonged cardiac prescriptions with repeats. It is hypothesized that the clinician-targeted educational outreach and reminders influenced these prescription patterns even when using non-intervention discharge prescriptions. However, barriers at the time of initial prescription fill further accounted for poor uptake of the intervention. Current prescription dispensation policies of the ODB program limit initial prescription dispensation to 30 days for new medications. However, it can be argued that the cardiac medications are not new, as they have already been initiated while participants in the study were inpatients (i.e., prior to discharge). To circumvent this policy limitation, educational outreach via personal emails and newsletters to community pharmacies in the study region was undertaken via the Ontario Pharmacy Association and the Ontario Pharmacy Evidence Network [23]. This outreach was intended to help ensure fidelity of the intervention when medications were dispensed at discharge. Furthermore, the standardized prolonged discharge prescription forms included a billing code that pharmacies could use to enable 90-day dispensation of new medications. Financial incentives are another potential barrier at the local pharmacy level. As per the ODB program [22], local pharmacies are reimbursed for up to five dispensations per patient in a 1-year period. Thus, prolonged dispensations would limit pharmacy revenues. These barriers at discharge and initial prescription fill likely accounted for the low 90-day dispensation proportions in the standardized prolonged discharge prescription forms plus education group (12%) and in the education-only group (9.5%). Unfortunately, this policy issue is not limited to Ontario [32]. Therefore, future evaluations of this intervention should focus on (1) restricting discharge prescriptions to an electronic health record system, which would enable control over prescribing practices and appropriate record keeping, thereby optimizing intervention fidelity within hospitals and (2) revising policies that presently limit long-term prescription dispensation at the pharmacy-level following hospital discharge.

As highlighted, poor intervention fidelity and low monthly numbers of patients in both intervention groups are key limitations of this study. Restriction of the study population to patients ≥ 65 years old to ensure completeness of prescription dispensation records (in the ODB database) is also a limitation that warrants consideration. Due to increased medical comorbidities and polypharmacy in older patients, the dispensation of medications may differ when compared to younger patients. For instance, the use of blister packs that limit the quantity of medications dispensed at one time is much more common for older versus younger patients. Unfortunately, random allocation of the intervention and control conditions across Ontario hospitals was not feasible. While we adjusted for several patient characteristics (including type of MI) to minimize confounding in secondary patient-level analyses, there are surely unmeasured confounders (due to non-randomized allocation) that could introduce bias into our estimated intervention effects within hospital groups and hinder between-group comparisons. Importantly, our analytic models controlled for pre-implementation (baseline) levels and trends in modeled outcomes when estimating the immediate and gradual effects of implemented interventions [33]. All measures to assess medication adherence have limitations. Although, novel methods for chemical adherence testing have been reported, these are best reserved for individual patients rather than the population level [34]. This study was designed to use pre-existing administrative datasets, therefore PDC was used and the threshold of ≥ 80% is associated with improved mortality in patients post-MI [10].

## Conclusions

This is the first non-randomized interventional study to evaluate an intervention to standardize discharge prescriptions to prolonged duration to improve long-term medication adherence among post-MI patients. Our findings, coupled with two large scale retrospective analyses of administrative data in the USA and Canada support further evaluation of this simple intervention to improve long-term adherence to cardiac medications [4, 18]. The risks and costs are small, but the potential for clinical benefit at the population level is significant. To ensure optimal fidelity, it is recommended that a similar intervention be implemented and evaluated in a larger population with fully electronic medical records coupled with policies to support the long-term dispensation of medications at the community pharmacy-level.

## Supplementary Information

**Additional file 1: Figure S1.** Monthly proportion of post-myocardial infarction patients age 65 and older with initial average prescription duration (i.e., days supplied at index fill) ≥90 days for cardiac medications – stratified by intervention group – from September 2015 to August 2018 in Ontario, Canada. *Notes*: Observed values are denoted by x, solid lines represent the fitted regression pre- and post-intervention trendlines for a given group, and hatched lines represent the projected pre-intervention trend assuming there was no intervention in that group (i.e., the counterfactual).

**Additional file 2: Table S1.** Baseline characteristics of post-myocardial infarction patients age 65 and older discharged home following a cardiac catheterization in Ontario, Canada from September 2015 to August 2018 – stratified by intervention group.

**Additional file 3: Table S2.** Results from patient-level segmented regression analyses estimating the relative immediate and gradual effects of study interventions on secondary medication-related outcomes among post-myocardial infarction patients age 65 and older in Ontario, Canada from September 2015 to August 2018.

**Additional file 4: Table S3.** Results from patient-level segmented regression analyses estimating the relative immediate and gradual effects of study interventions on secondary outcomes capturing health care utilization and clinical outcomes among post-myocardial infarction patients age 65 and older in Ontario, Canada from September 2015 to August 2018.

**Additional file 5: Table S4.** Proportion of patients with a prolonged discharge prescription for ≥90 days with 3 repeats by cardiac medication class during the 12-month intervention period based on hospital chart data. *Notes:* a ≥ 90 days supplied on average across cardiac medication classes with a discharge prescription without consideration of number of repeats on prescription(s).

## Data Availability

The dataset from this study is held securely in coded form at ICES. While data sharing agreements prohibit ICES from making the dataset publicly available, access may be granted to those who meet pre-specified criteria for confidential access, available at www.ices.on.ca/DAS. The full dataset creation plan and underlying analytic code are available from the authors upon request, understanding that the computer programs may rely upon coding templates or macros that are unique to ICES and are therefore either inaccessible or may require modification.

## Abbreviations

ACE: Angiotensin-converting enzyme inhibitor
ARB: Angiotensin receptor blocker
CABG: Coronary artery bypass graft surgery
CAD: Coronary artery disease
CI: Confidence interval
MI: Myocardial infarction
MIPAD: Myocardial Infarction Prescription Duration Study
NSTEMI: Non-ST-elevated MI
ODB: Ontario Drug Benefit
PCI: Percutaneous coronary intervention
PDC: Proportion of days covered
STEMI: ST-elevated MI
