# Supplementary material for: Length of initial prescription at hospital discharge and long-term medication adherence for elderly, post-myocardial infarction patients: a population-based interrupted time series study
Source: BMC Med. 2022 Jun 21;20:213. doi: 10.1186/s12916-022-02401-5 (PMC9210591; doi:10.1186/s12916-022-02401-5)

Additional File 1: Fig.S. Monthly proportion of post-myocardial infarction patients age 65 and older with initial average prescription duration (i.e., days supplied at index fill) ≥90 days for cardiac medications – stratified by intervention group – from September 2015 to August 2018 in Ontario, Canada.

Notes: Observed values are denoted by x, solid lines represent the fitted regression pre- and post-intervention trendlines for a given group, and hatched lines represent the projected pre-intervention trend assuming there was no intervention in that group (i.e., the counterfactual).

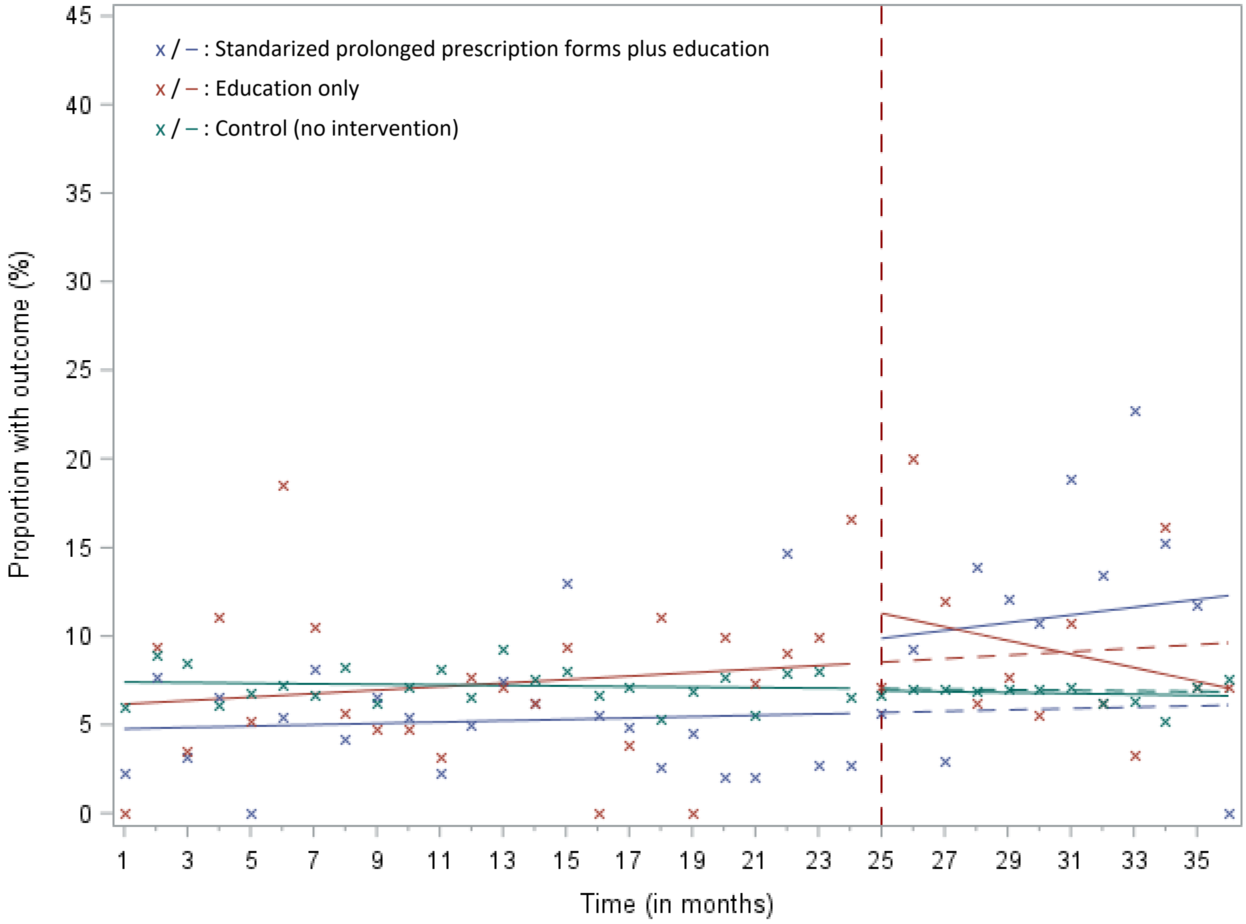

Supplement: Supplementary file 1 — Additional file 1: Figure S1. Monthly proportion of post-myocardial infarction patients age 65 and older with initial average prescription duration (i.e., days supplied at index fill) ≥90 days for cardiac medications – stratified by intervention group – from September 2015 to August 2018 in Ontario, Canada. Notes: Observed values are denoted by x, solid lines represent the fitted regression pre- and post-intervention trendlines for a given group, and hatched lines represent the projected pre-intervention trend assuming there was no intervention in that group (i.e., the counterfactual). [file 12916_2022_2401_MOESM1_ESM.pdf]
