# Supplementary material for: Length of initial prescription at hospital discharge and long-term medication adherence for elderly, post-myocardial infarction patients: a population-based interrupted time series study
Source: BMC Med. 2022 Jun 21;20:213. doi: 10.1186/s12916-022-02401-5 (PMC9210591; doi:10.1186/s12916-022-02401-5)
Supplement: Supplementary file 2 — Additional file 2: Table S1. Baseline characteristics of post-myocardial infarction patients age 65 and older discharged home following a cardiac catheterization in Ontario, Canada from September 2015 to August 2018 – stratified by intervention group. [file 12916_2022_2401_MOESM2_ESM.pdf]

Additional File 2: TableS1. Baseline characteristics of post-myocardial infarction patients age 65 and older discharged home following a cardiac catheterization in Ontario, Canada from September 2015 to August 2018 – stratified by intervention group.

		Intervention group		
	Overall	Standardized prolonged discharge prescription forms plus education	Education only	Control
Patient-level characteristics	N=20,896 (149 sites)	N=1,414 (2 sites)	N=926 (4 sites)	N=18,556 (143 sites)
Female, n (%)	7,062 (33.8%)	432 (30.6%)	359 (38.8%)	6,271 (33.8%)
Age (mean±SD)	75.2±7.1	74.6±6.9	75.6±7.0	75.2±7.1
Primary reason for referral=STEMI, n(%)	6,371 (30.5%)	455 (32.2%)	212 (22.9%)	5,704 (30.7%)
Prior myocardial infarction=yes, n(%)	5,281 (25.3%)	381 (26.9%)	254 (27.4%)	4,646[a] (25.0%)
Prior cardiac medication use=yes[b], n(%)	14,665 (70.2%)	1,030 (72.8%)	675 (72.9%)	12,960 (69.8%)

Notes: STEMI = ST-elevated myocardial infarction. Percentages are column percentages. Baseline characteristics ascertained as of discharge date according to linked Discharge Abstract Database claims detailing an inpatient episode of care overlapping with index catheterization procedure date.

[a] 10 missing values (<0.1% overall) observed for prior myocardial infarction variable among control group. Column percentage for this cell calculated after excluding missing values from denominator.

[b] Any dispensation(s) for a statin, beta blocker, angiotensin system inhibitor, or secondary antiplatelet in 120 days prior to discharge date.
