# Supplementary material for: Length of initial prescription at hospital discharge and long-term medication adherence for elderly, post-myocardial infarction patients: a population-based interrupted time series study
Source: BMC Med. 2022 Jun 21;20:213. doi: 10.1186/s12916-022-02401-5 (PMC9210591; doi:10.1186/s12916-022-02401-5)
Supplement: Supplementary file 3 — Additional file 3: Table S2. Results from patient-level segmented regression analyses estimating the relative immediate and gradual effects of study interventions on secondary medication-related outcomes among post-myocardial infarction patients age 65 and older in Ontario, Canada from September 2015 to August 2018. [file 12916_2022_2401_MOESM3_ESM.pdf]

Additional File 3: TableS2. Results from patient-level segmented regression analyses estimating the relative immediate and gradual effects of study interventions on secondary medication-related outcomes among post-myocardial infarction patients age 65 and older in Ontario, Canada from September 2015 to August 2018.

Intervention	Parameter	Exp(Estimate)[a] (95% CI)	P value
**Number of cardiac medications dispensed at index fill (count)**			
**Standardized prolonged discharge prescription forms plus education (2 sites, 1,414 patients)**	Pre-intervention slope (secular trend, per month)	1.00 (1.00, 1.01)	.31
	Change in level post-intervention (immediate effect)	1.04 (0.90, 1.19)	.60
	Change in trend post-intervention (gradual effect, per month)	0.99 (0.97, 1.01)	.32
**Education only (4 sites, 926 patients)**	Pre-intervention slope (secular trend, per month)	1.00 (0.99, 1.01)	.82
	Change in level post-intervention (immediate effect)	0.97 (0.83, 1.14)	.75
	Change in trend post-intervention (gradual effect, per month)	1.00 (0.98, 1.02)	.99
**Control (26 sites, 14,336 patients[b])**	Pre-intervention slope (secular trend, per month)	1.00 (1.00, 1.00)	.29
	Change in level post-intervention (immediate effect)	0.99 (0.95, 1.03)	.69
	Change in trend post-intervention (gradual effect, per month)	1.00 (0.99, 1.00)	.75
**Beta blocker adherence at 1 year (dichotomous)**			
**Standardized prolonged discharge prescription forms plus education (2 sites, 1016 patients)**	Pre-intervention slope (secular trend, per month)	1.00 (0.97, 1.03)	.94
	Change in level post-intervention (immediate effect)	0.88 (0.45, 1.72)	.71
	Change in trend post-intervention (gradual effect, per month)	1.02 (0.94, 1.11)	.61
**Education only (4 sites, 687 patients)**	Pre-intervention slope (secular trend, per month)	1.01 (0.97, 1.04)	.67
	Change in level post-intervention (immediate effect)	1.09 (0.47, 2.55)	.84
	Change in trend post-intervention (gradual effect, per month)	0.94 (0.85, 1.04)	.21
**Control (26 sites, 10,244 patients[b])**	Pre-intervention slope (secular trend, per month)	1.00 (0.99, 1.01)	.45
	Change in level post-intervention (immediate effect)	1.03 (0.83, 1.27)	.81
	Change in trend post-intervention (gradual effect, per month)	1.00 (0.97, 1.03)	.97
**Angiotensin system inhibitor adherence at 1 year (dichotomous)**			
**Standardized prolonged discharge prescription forms plus education (2 sites, 731 patients)**	Pre-intervention slope (secular trend, per month)	0.97 (0.93, 1.00)	.04
	Change in level post-intervention (immediate effect)	1.13 (0.52, 2.46)	.77
	Change in trend post-intervention (gradual effect, per month)	1.08 (0.97, 1.19)	.16
**Education only (4 sites, 534 patients)**	Pre-intervention slope (secular trend, per month)	0.99 (0.95, 1.03)	.61
	Change in level post-intervention	0.83 (0.35, 1.96)	.67

	(immediate effect)		
	Change in trend post-intervention (gradual effect, per month)	1.02 (0.92, 1.14)	.68
**Control (26 sites, 8,528 patients[b])**	Pre-intervention slope (secular trend, per month)	0.99 (0.98, 1.00)	.17
	Change in level post-intervention (immediate effect)	1.19 (0.93, 1.51)	.16
	Change in trend post-intervention (gradual effect, per month)	0.97 (0.95, 1.00)	.07
**Statin adherence at 1 year (dichotomous)**			
**Standardized prolonged discharge prescription forms plus education (2 sites, 1,146 patients)**	Pre-intervention slope (secular trend, per month)	1.00 (0.97, 1.03)	.80
	Change in level post-intervention (immediate effect)	1.27 (0.58, 2.78)	.55
	Change in trend post-intervention (gradual effect, per month)	1.00 (0.91, 1.11)	.94
**Education only (4 sites, 761 patients)**	Pre-intervention slope (secular trend, per month)	1.04 (1.00, 1.08)	.05
	Change in level post-intervention (immediate effect)	0.48 (0.19, 1.21)	.12
	Change in trend post-intervention (gradual effect, per month)	1.05 (0.93, 1.18)	.44
**Control (26 sites, 11,631 patients[b])**	Pre-intervention slope (secular trend, per month)	1.01 (1.00, 1.02)	.12
	Change in level post-intervention (immediate effect)	0.96 (0.76, 1.22)	.76
	Change in trend post-intervention (gradual effect, per month)	0.99 (0.96, 1.02)	.43
**Secondary antiplatelet adherence at 1 year (dichotomous)**			
**Standardized prolonged discharge prescription forms plus education (2 sites, 984 patients)**	Pre-intervention slope (secular trend, per month)	1.02 (0.99, 1.05)	.17
	Change in level post-intervention (immediate effect)	0.80 (0.40, 1.57)	.51
	Change in trend post-intervention (gradual effect, per month)	1.03 (0.94, 1.12)	.56
**Education only (4 sites, 784 patients)**	Pre-intervention slope (secular trend, per month)	1.02 (0.99, 1.06)	.11
	Change in level post-intervention (immediate effect)	0.69 (0.31, 1.53)	.37
	Change in trend post-intervention (gradual effect, per month)	1.08 (0.97, 1.20)	.18
**Control (26 sites, 11,861 patients[b])**	Pre-intervention slope (secular trend, per month)	1.00 (0.99, 1.01)	.49
	Change in level post-intervention (immediate effect)	1.02 (0.82, 1.28)	.84
	Change in trend post-intervention (gradual effect, per month)	1.01 (0.98, 1.04)	.61
**Beta blocker persistence at 1 year (dichotomous)**			
**Standardized prolonged discharge prescription forms plus education (2 sites, 1,016 patients)**	Pre-intervention slope (secular trend, per month)	1.00 (0.97, 1.02)	.75
	Change in level post-intervention (immediate effect)	0.77 (0.42, 1.42)	.41
	Change in trend post-intervention (gradual effect, per month)	1.06 (0.98, 1.14)	.15
**Education only (4 sites, 687 patients)**	Pre-intervention slope (secular trend, per month)	1.02 (0.99, 1.05)	.22

	Change in level post-intervention (immediate effect)	0.96 (0.44, 2.08)	.92
	Change in trend post-intervention (gradual effect, per month)	0.94 (0.85, 1.03)	.16
**Control (26 sites, 10,244 patients[b])**	Pre-intervention slope (secular trend, per month)	1.00 (0.99, 1.01)	.48
	Change in level post-intervention (immediate effect)	1.09 (0.89, 1.32)	.41
	Change in trend post-intervention (gradual effect, per month)	0.99 (0.97, 1.02)	.48
**Angiotensin system inhibitor persistence at 1 year (dichotomous)**			
**Standardized prolonged discharge prescription forms plus education (2 sites, 731 patients)**	Pre-intervention slope (secular trend, per month)	0.98 (0.95, 1.01)	.23
	Change in level post-intervention (immediate effect)	0.99 (0.48, 2.03)	.98
	Change in trend post-intervention (gradual effect, per month)	1.05 (0.96, 1.15)	.32
**Education only (4 sites, 534 patients)**	Pre-intervention slope (secular trend, per month)	0.98 (0.95, 1.02)	.32
	Change in level post-intervention (immediate effect)	0.96 (0.44, 2.13)	.93
	Change in trend post-intervention (gradual effect, per month)	1.05 (0.95, 1.16)	.37
**Control (26 sites, 8,528 patients[b])**	Pre-intervention slope (secular trend, per month)	1.00 (0.99, 1.01)	.39
	Change in level post-intervention (immediate effect)	1.10 (0.89, 1.37)	.37
	Change in trend post-intervention (gradual effect, per month)	0.98 (0.96, 1.01)	.17
**Statin persistence at 1 year (dichotomous)**			
**Standardized discharge prescription forms plus education (2 sites, 1,146 patients)**	Pre-intervention slope (secular trend, per month)	1.01 (0.98, 1.04)	.43
	Change in level post-intervention (immediate effect)	0.86 (0.45, 1.64)	.65
	Change in trend post-intervention (gradual effect, per month)	1.01 (0.94, 1.10)	.73
**Education only (4 sites, 761 patients)**	Pre-intervention slope (secular trend, per month)	1.04 (1.01, 1.07)	.02
	Change in level post-intervention (immediate effect)	0.69 (0.31, 1.54)	.37
	Change in trend post-intervention (gradual effect, per month)	0.97 (0.88, 1.07)	.54
**Control (26 sites, 11,631 patients[b])**	Pre-intervention slope (secular trend, per month)	1.01 (1.00, 1.02)	<.001
	Change in level post-intervention (immediate effect)	0.90 (0.73, 1.09)	.28
	Change in trend post-intervention (gradual effect, per month)	0.99 (0.96, 1.01)	.33
**Secondary antiplatelet persistence at 1 year (dichotomous)**			
**Standardized prolonged discharge prescription forms plus education (2 sites, 984 patients)**	Pre-intervention slope (secular trend, per month)	1.01 (0.98, 1.04)	.45
	Change in level post-intervention (immediate effect)	0.88 (0.47, 1.64)	.68
	Change in trend post-intervention (gradual effect, per month)	1.02 (0.95, 1.11)	.55
**Education only**	Pre-intervention slope	1.03 (1.00, 1.05)	.08

**(4 sites, 784 patients)**	(secular trend, per month)		
	Change in level post-intervention (immediate effect)	0.74 (0.36, 1.50)	.40
	Change in trend post-intervention (gradual effect, per month)	1.01 (0.92, 1.10)	.90
**Control (26 sites, 11,861 patients[b])**	Pre-intervention slope (secular trend, per month)	1.00 (0.99, 1.01)	.63
	Change in level post-intervention (immediate effect)	1.19 (0.98, 1.46)	.08
	Change in trend post-intervention (gradual effect, per month)	1.00 (0.98, 1.03)	.94
**Mean days supplied ≥90 days per cardiac medication at index fill[c] (dichotomous)**			
**Standardized prolonged discharge prescription forms plus education (2 sites, 1,414 patients)**	Pre-intervention slope (secular trend, per month)	1.00 (0.96, 1.05)	.86
	Change in level post-intervention (immediate effect)	1.77 (0.75, 4.17)	.19
	Change in trend post-intervention (gradual effect, per month)	1.04 (0.95, 1.14)	.41
**Education only (4 sites, 784 patients)**	Pre-intervention slope (secular trend, per month)	1.01 (0.97, 1.05)	.65
	Change in level post-intervention (immediate effect)	1.63 (0.63, 4.22)	.31
	Change in trend post-intervention (gradual effect, per month)	0.96 (0.85, 1.08)	.48
**Control (26 sites, 14,366 patients[b])**	Pre-intervention slope (secular trend, per month)	1.00 (0.99, 1.01)	.74
	Change in level post-intervention (immediate effect)	0.93 (0.71, 1.23)	.61
	Change in trend post-intervention (gradual effect, per month)	1.01 (0.97, 1.04)	.66
**Mean days supplied ≥90 days for beta blocker at index fill[c] (dichotomous)**			
**Standardized prolonged discharge prescription forms plus education (2 sites, 1,016 patients)**	Pre-intervention slope (secular trend, per month)	1.01 (0.98, 1.05)	.48
	Change in level post-intervention (immediate effect)	1.59 (0.70, 3.62)	.27
	Change in trend post-intervention (gradual effect, per month)	0.98 (0.89, 1.08)	.71
**Education only (4 sites, 687 patients)**	Pre-intervention slope (secular trend, per month)	0.96 (0.92, 1.00)	.06
	Change in level post-intervention (immediate effect)	1.32 (0.46, 3.77)	.60
	Change in trend post-intervention (gradual effect, per month)	1.10 (0.97, 1.24)	.13
**Control (26 sites, 10,244 patients[b])**	Pre-intervention slope (secular trend, per month)	1.00 (0.99, 1.01)	.78
	Change in level post-intervention (immediate effect)	0.82 (0.63, 1.07)	.14
	Change in trend post-intervention (gradual effect, per month)	1.01 (0.98, 1.05)	.38
**Mean days supplied ≥90 days for angiotensin system inhibitor at index fill[c] (dichotomous)**			
**Standardized prolonged discharge prescription forms plus education (2 sites, 731 patients)**	Pre-intervention slope (secular trend, per month)	0.97 (0.94, 1.01)	.13
	Change in level post-intervention (immediate effect)	3.48 (1.51, 8.03)	<.001
	Change in trend post-intervention (gradual effect, per month)	0.97 (0.87, 1.07)	.51

**Education only (4 sites, 534 patients)**	Pre-intervention slope (secular trend, per month)	1.00 (0.96, 1.05)	.92
	Change in level post-intervention (immediate effect)	2.45 (0.92, 6.56)	.08
	Change in trend post-intervention (gradual effect, per month)	0.88 (0.77, 1.00)	.06
**Control (26 sites, 8,528 patients[b])**	Pre-intervention slope (secular trend, per month)	1.00 (0.99, 1.01)	.77
	Change in level post-intervention (immediate effect)	0.94 (0.72, 1.22)	.64
	Change in trend post-intervention (gradual effect, per month)	1.01 (0.98, 1.04)	.52
**Mean days supplied ≥90 days for statin at index fill[c] (dichotomous)**			
**Standardized prolonged discharge prescription forms plus education (2 sites, 1,146 patients)**	Pre-intervention slope (secular trend, per month)	1.02 (0.99, 1.05)	.26
	Change in level post-intervention (immediate effect)	1.92 (1.04, 3.57)	.04
	Change in trend post-intervention (gradual effect, per month)	0.95 (0.88, 1.03)	.21
**Education only (4 sites, 761 patients)**	Pre-intervention slope (secular trend, per month)	1.02 (0.99, 1.05)	.22
	Change in level post-intervention (immediate effect)	0.92 (0.42, 2.04)	.85
	Change in trend post-intervention (gradual effect, per month)	0.98 (0.89, 1.08)	.69
**Control (26 sites, 11,631 patients[b])**	Pre-intervention slope (secular trend, per month)	1.00 (0.99, 1.01)	.89
	Change in level post-intervention (immediate effect)	0.97 (0.78, 1.19)	.74
	Change in trend post-intervention (gradual effect, per month)	1.00 (0.98, 1.03)	.80
**Mean days supplied ≥90 days for secondary antiplatelet at index fill[c] (dichotomous)**			
**Standardized prolonged discharge prescription forms plus education (2 sites, 984 patients)**	Pre-intervention slope (secular trend, per month)	1.00 (0.96, 1.05)	.87
	Change in level post-intervention (immediate effect)	1.44 (0.58, 3.58)	.43
	Change in trend post-intervention (gradual effect, per month)	1.04 (0.94, 1.15)	.48
**Education only (4 sites, 784 patients)**	Pre-intervention slope (secular trend, per month)	1.01 (0.96, 1.06)	.66
	Change in level post-intervention (immediate effect)	2.06 (0.77, 5.52)	.15
	Change in trend post-intervention (gradual effect, per month)	0.92 (0.81, 1.05)	.22
**Control (26 sites, 11,861 patients[b])**	Pre-intervention slope (secular trend, per month)	1.00 (0.99, 1.01)	.86
	Change in level post-intervention (immediate effect)	0.94 (0.70, 1.27)	.70
	Change in trend post-intervention (gradual effect, per month)	1.00 (0.97, 1.04)	.87

Notes: CI = confidence interval.

[a] All estimates adjusted for site (as fixed effect for both intervention group-specific models; as random effect (site-specific intercept) for control analysis) and the following patient covariates: age, sex, primary reason for index cardiac catheterization (STEMI vs NSTEMI); prior myocardial infarction; and an indicator of prior cardiac medication use. The effect measure calculated as exp(estimate) depends on the type of outcome, i.e., exp(estimate)=odds ratio for a dichotomous outcome, exp(estimate)=rate ratio for a count outcome, and exp(estimate)= hazard ratio for a time-to-event outcomes.

[b] To facilitate model convergence, the control series was reduced from 143 to 26 sites after restricting to sites with a minimum of 180 patients over the 36-month study window. A total of 14,334 patients were discharged across 26 sites in reduced control series; however, patients with missing information for the prior cardiac medication use covariate (n=8) were excluded from regression analyses.

[c] Index fill for cardiac medications occurred within 7 days of discharge for all patients in the study.
