# Supplementary material for: Length of initial prescription at hospital discharge and long-term medication adherence for elderly, post-myocardial infarction patients: a population-based interrupted time series study
Source: BMC Med. 2022 Jun 21;20:213. doi: 10.1186/s12916-022-02401-5 (PMC9210591; doi:10.1186/s12916-022-02401-5)
Supplement: Supplementary file 4 — Additional file 4: Table S3. Results from patient-level segmented regression analyses estimating the relative immediate and gradual effects of study interventions on secondary outcomes capturing health care utilization and clinical outcomes among post-myocardial infarction patients age 65 and older in Ontario, Canada from September 2015 to August 2018. [file 12916_2022_2401_MOESM4_ESM.pdf]

Additional File 4: TableS3. Results from patient-level segmented regression analyses estimating the relative immediate and gradual effects of study interventions on secondary outcomes capturing health care utilization and clinical outcomes among post-myocardial infarction patients age 65 and older in Ontario, Canada from September 2015 to August 2018.

Intervention	Parameter	Exp(Estimate)[a] (95% CI)	P value
**Frequency of outpatient primary care visits at 1 year (count)**			
**Standardized prolonged discharge prescription forms plus education (2 sites, 1,414 patients)**	Pre-intervention slope (secular trend, per month)	1.00 (0.99, 1.01)	.72
	Change in level post-intervention (immediate effect)	1.11 (0.91, 1.34)	.30
	Change in trend post-intervention (gradual effect, per month)	0.97 (0.95, 1.00)	.03
**Education only (4 sites, 926 patients)**	Pre-intervention slope (secular trend, per month)	1.00 (0.99, 1.00)	.32
	Change in level post-intervention (immediate effect)	1.01 (0.81, 1.26)	.96
	Change in trend post-intervention (gradual effect, per month)	0.99 (0.96, 1.02)	.42
**Control (26 sites, 14,336 patients[b])**	Pre-intervention slope (secular trend, per month)	1.00 (1.00, 1.00)	.35
	Change in level post-intervention (immediate effect)	1.00 (0.94, 1.05)	.91
	Change in trend post-intervention (gradual effect, per month)	1.00 (0.99, 1.01)	.69
**Frequency of outpatient cardiology visits at 1 year (count)**			
**Standardized prolonged discharge prescription forms plus education (2 sites, 1414 patients)**	Pre-intervention slope (secular trend, per month)	1.00 (1.00, 1.01)	.33
	Change in level post-intervention (immediate effect)	0.99 (0.81, 1.22)	.94
	Change in trend post-intervention (gradual effect, per month)	1.00 (0.98, 1.03)	.90
**Education only (4 sites, 926 patients)**	Pre-intervention slope (secular trend, per month)	1.00 (0.99, 1.01)	.53
	Change in level post-intervention (immediate effect)	0.95 (0.72, 1.26)	.72
	Change in trend post-intervention (gradual effect, per month)	1.00 (0.97, 1.04)	.90
**Control (26 sites, 14,336 patients[b])**	Pre-intervention slope (secular trend, per month)	1.00 (1.00, 1.01)	.07
	Change in level post-intervention (immediate effect)	1.02 (0.96, 1.09)	.52
	Change in trend post-intervention (gradual effect, per month)	1.00 (0.99, 1.01)	.87
**Death (time-to-event [in days])**			
**Standardized prolonged discharge prescription forms plus education (2 sites, 1,414 patients, 89 events)**	Pre-intervention slope (secular trend, per month)	0.99 (0.95, 1.02)	.53
	Change in level post-intervention (immediate effect)	0.44 (0.14, 1.35)	.15
	Change in trend post-intervention (gradual effect, per month)	1.12 (0.99, 1.27)	.08
**Education only (4 sites, 926 patients, 79 events)**	Pre-intervention slope (secular trend, per month)	0.97 (0.93, 1.01)	.15
	Change in level post-intervention	1.27 (0.46, 3.46)	.64

	(immediate effect)		
	Change in trend post-intervention (gradual effect, per month)	1.08 (0.97, 1.21)	.18
**Control (26 sites, 14,336 patients[b], 1,038 events)**	Pre-intervention slope (secular trend, per month)	1.00 (0.98, 1.01)	.42
	Change in level post-intervention (immediate effect)	1.10 (0.85, 1.43)	.48
	Change in trend post-intervention (gradual effect, per month)	1.01 (0.98, 1.04)	.65
**Cardiovascular-related hospitalization (time-to-event [in days])**			
**Standardized prolonged discharge prescription forms plus education (2 sites, 1,414 patients, 404 events)**	Pre-intervention slope (secular trend, per month)	1.00 (0.98, 1.02)	.98
	Change in level post-intervention (immediate effect)	1.01 (0.66, 1.56)	.95
	Change in trend post-intervention (gradual effect, per month)	1.02 (0.97, 1.07)	.54
**Education only (4 sites, 926 patients, 301 events)**	Pre-intervention slope (secular trend, per month)	1.00 (0.98, 1.02)	.97
	Change in level post-intervention (immediate effect)	1.14 (0.70, 1.85)	.61
	Change in trend post-intervention (gradual effect, per month)	1.00 (0.94, 1.06)	.99
**Control (26 sites, 14,336 patients[b], 4,304 events)**	Pre-intervention slope (secular trend, per month)	1.00 (1.00, 1.01)	.74
	Change in level post-intervention (immediate effect)	1.07 (0.94, 1.21)	.31
	Change in trend post-intervention (gradual effect, per month)	1.00 (0.98, 1.01)	.80
**Acute myocardial infarction-related hospitalization (time-to-event [in days])**			
**Standardized prolonged discharge prescription forms plus education (2 sites, 1,414 patients, 130 events)**	Pre-intervention slope (secular trend, per month)	1.01 (0.97, 1.04)	.72
	Change in level post-intervention (immediate effect)	0.88 (0.41, 1.88)	.73
	Change in trend post-intervention (gradual effect, per month)	1.04 (0.95, 1.13)	.41
**Education only (4 sites, 926 patients, 108 events)**	Pre-intervention slope (secular trend, per month)	1.01 (0.98, 1.04)	.59
	Change in level post-intervention (immediate effect)	1.13 (0.51, 2.50)	.77
	Change in trend post-intervention (gradual effect, per month)	0.96 (0.87, 1.06)	.45
**Control (26 sites, 14,336 patients[b], 1,325 events)**	Pre-intervention slope (secular trend, per month)	1.00 (0.99, 1.01)	.39
	Change in level post-intervention (immediate effect)	1.13 (0.90, 1.42)	.30
	Change in trend post-intervention (gradual effect, per month)	1.01 (0.98, 1.03)	.70
**Stroke-related hospitalization (time-to-event [in days])**			
**Standardized prolonged discharge prescription forms plus education (2 sites, 1,414 patients, 20 events)**	Pre-intervention slope (secular trend, per month)	1.01 (0.94, 1.09)	.79
	Change in level post-intervention (immediate effect)	0.43 (0.05, 3.86)	.45
	Change in trend post-intervention (gradual effect, per month)	1.09 (0.85, 1.40)	.50
**Education only (4 sites, 926 patients, 13 events)**	Pre-intervention slope (secular trend, per month)	0.94 (0.84, 1.05)	.29

	Change in level post-intervention (immediate effect)	8.02 (0.81, 79.02)	.07
	Change in trend post-intervention (gradual effect, per month)	0.94 (0.73, 1.23)	.67
**Control (26 sites, 14,336 patients[b], 175 events)**	Pre-intervention slope (secular trend, per month)	0.98 (0.96, 1.01)	.18
	Change in level post-intervention (immediate effect)	2.19 (1.20, 3.99)	.01
	Change in trend post-intervention (gradual effect, per month)	0.92 (0.85, 1.00)	.05
**Coronary revascularization procedure (time-to-event [in days])**			
**Standardized prolonged discharge prescription forms plus education (2 sites, 1,414 patients, 115 events)**	Pre-intervention slope (secular trend, per month)	1.00 (0.97, 1.03)	.88
	Change in level post-intervention (immediate effect)	1.28 (0.58, 2.82)	.54
	Change in trend post-intervention (gradual effect, per month)	0.97 (0.88, 1.07)	.60
**Education only (4 sites, 926 patients, 85 events)**	Pre-intervention slope (secular trend, per month)	1.03 (0.99, 1.07)	.12
	Change in level post-intervention (immediate effect)	0.52 (0.20, 1.32)	.16
	Change in trend post-intervention (gradual effect, per month)	1.02 (0.91, 1.14)	.78
**Control (26 sites, 14,336 patients[b], 1,471 events)**	Pre-intervention slope (secular trend, per month)	1.01 (1.00, 1.02)	.16
	Change in level post-intervention (immediate effect)	1.12 (0.90, 1.38)	.30
	Change in trend post-intervention (gradual effect, per month)	1.00 (0.97, 1.02)	.81
**Cardiac catheterization procedure (time-to-event [in days])**			
**Standardized prolonged discharge prescription forms plus education (2 sites, 1,414 patients, 132 events)**	Pre-intervention slope (secular trend, per month)	0.98 (0.95, 1.01)	.26
	Change in level post-intervention (immediate effect)	2.31 (1.13, 4.71)	.02
	Change in trend post-intervention (gradual effect, per month)	0.97 (0.89, 1.05)	.45
**Education only (4 sites, 926 patients, 93 events)**	Pre-intervention slope (secular trend, per month)	1.01 (0.98, 1.05)	.48
	Change in level post-intervention (immediate effect)	0.68 (0.28, 1.65)	.39
	Change in trend post-intervention (gradual effect, per month)	1.03 (0.93, 1.15)	.53
**Control (26 sites, 14,336 patients[b], 1,808 events)**	Pre-intervention slope (secular trend, per month)	1.00 (0.99, 1.01)	.46
	Change in level post-intervention (immediate effect)	1.24 (1.03, 1.50)	.03
	Change in trend post-intervention (gradual effect, per month)	0.99 (0.96, 1.01)	.26

Notes: CI = confidence interval.

[a] All estimates adjusted for site (as fixed effect for both intervention group-specific models; as random effect (site-specific intercept) for control analysis) and the following patient covariates: age, sex, primary reason for index cardiac catheterization (STEMI vs NSTEMI); prior myocardial infarction; and an indicator of prior cardiac medication use. The effect measure calculated as exp(estimate) depends on the type of outcome, i.e., exp(estimate)=odds ratio for a dichotomous outcome, exp(estimate)=rate ratio for a count outcome, and exp(estimate)= hazard ratio for a time-to-event outcomes.

[b] To facilitate model convergence, the control series was reduced from 143 to 26 sites after restricting to sites with a minimum of 180 patients over the 36-month study window. A total of 14,334 patients were discharged across 26 sites in reduced control series; however, patients with missing information for the prior cardiac medication use covariate (n=8) were excluded from regression analyses.

[c] Index fill for cardiac medications occurred within 7 days of discharge for all patients in the study.
