# Supplementary material for: Length of initial prescription at hospital discharge and long-term medication adherence for elderly, post-myocardial infarction patients: a population-based interrupted time series study
Source: BMC Med. 2022 Jun 21;20:213. doi: 10.1186/s12916-022-02401-5 (PMC9210591; doi:10.1186/s12916-022-02401-5)
Supplement: Supplementary file 5 — Additional file 5: Table S4. Proportion of patients with a prolonged discharge prescription for ≥90 days with 3 repeats by cardiac medication class during the 12-month intervention period based on hospital chart data. Notes: a ≥ 90 days supplied on average across cardiac medication classes with a discharge prescription without consideration of number of repeats on prescription(s). [file 12916_2022_2401_MOESM5_ESM.pdf]

Additional File 5: TableS4. Proportion of patients with a prolonged discharge prescription for ≥90 days with 3 repeats by cardiac medication class during the 12-month intervention period based on hospital chart data. *Notes:* [a] ≥90 days supplied on average across cardiac medication classes with a discharge prescription without consideration of number of repeats on prescription(s).

	**Standardized prolonged discharge prescription forms plus education**	**Education only**
Average (across classes)[a]	34/41 (83%)	34/51 (67%)
Statins	29/40 (73%)	25/41 (61%)
Beta blockers	23/33 (70%)	24/43 (56%)
Angiotensin system inhibitors	23/32 (72%)	17/32 (53%)
Secondary antiplatelets	34/39 (87%)	33/48 (69%)
